# Metabolome and transcriptome profiling reveal regulatory network and mechanism of flavonoid biosynthesis during color formation of *Dioscorea cirrhosa* L.

**DOI:** 10.7717/peerj.13659

**Published:** 2022-07-04

**Authors:** Lin Yan, Haijun Yang, Qiang Ye, Zhihua Huang, Hongying Zhou, Dafang Cui

**Affiliations:** 1 College of Forestry and Landscape Architecture, South China Agricultural University, Guangzhou, Guangdong, China; 2 Center of Experimental Teaching for Common Basic Courses, South China Agricultural University, Guangzhou, Guangdong, China; 3 Shenzhen Liangzi Fashion Industeial Co. Ltd., Shenzhen, Guangdong, China; 4 Shenzhen Tianyi Xunyuan Ecological Culture Investment Co.Ltd., Shenzhen, Guangdong, China

**Keywords:** Flavonoids, Plant pigments, Proanthocyanidins, Metabolome, Transcriptome, *Dioscorea cirrhosa*

## Abstract

*Dioscorea cirrhosa* is a plant that is used as a dye as well as in medicine. Many metabolites with pharmacological activity exist in the tubers of *D. cirrhosa*. However, little is known about the mechanism regulating biosynthesis in these metabolites. In this study, transcriptome and metabolome profiling were performed in four color tubers. A total of 531 metabolites, including 62 flavonoids, were identified. Epicatechin and proanthocyanin B2 were the key metabolites that exhibited high content levels in the four tubers. These metabolites were divided into nine classes with distinct change patterns. A total of 22,865 differentially expressed genes (DEGs) were identified by transcriptome analysis. Among these DEGs, we identified 67 candidate genes related to the flavonoid biosynthesis pathway and three genes that played pivotal roles in proanthocyanin (PA) synthesis. A weighted gene co-expression network analysis (WGCNA) revealed that the two modules, “MEblue” and “MEblack,” were two key gene sets strongly associated with phenylpropanoid and flavonoid biosynthesis. We also found that the plant hormone signal transduction biological process exhibited activity in the late stage of tuber color formation. Additionally, we identified 37 hub transcript factors related to flavonoid biosynthesis, of which 24 were found to be highly associated with flavonoid pathway genes. In addition to the MYB-bHLH-WD40 (MBW) genes, we found that the plant hormone gene families exhibited high expression levels. This study provides a reference for understanding the synthesis of *D. cirrhosa* tuber metabolites at the molecular level and provides a foundation for the further development of *D. cirrhosa* related plant pigments as well as its further use in the pharmaceutical industry.

## Introduction

*Dioscorea cirrhosa* is a small bush that grows naturally on mountain slopes and forest edges. This species is mainly distributed in tropical and subtropical areas (Bechtold & Mussak, 2009). Its mature tubers have a unique red color that are rich in polyphenols, such as epicatechin, and catechins (Hsu, Nonaka & Nishioka, 1985). Modern medical research has shown that the tubers have an astringent effect on gastrointestinal bleeding, inhibit the reproduction of *Helicobacter pylori*, and promote the healing of ulcer surfaces (Guyot et al., 1999). Additionally, the extract of the pigments in the tubers can be used as a natural plant dye (Cheng et al., 2010). Metal ion treatment enhances the adsorption of these pigments into silk proteins producing dark and lustrous silk fabric (Cheng et al., 2010; Yang, Guan & Tang, 2018).

The molecular genetic mechanism related to plant color has always been a hot topic. Flavonoids, including anthocyanins, isoflavones, flavanones, flavonols, and chalcones, perform functions in pigment accumulation and protect plants against UV light and pathogens (Harborne & Williams, 2000). The flavonoid biosynthesis pathway and the regulation of gene expression by transcription factors are both related to color phenotype. In wolfberry plants, the abnormal expression of a basic helix-loop-helix protein (bHLH), *LrAN1b,* is highly correlated to anthocyanin accumulation and results in the white fruit phenotype of *Lycium ruthenicum* Murray (Li et al., 2020). The UDP glucose-flavonoid 3-o-glcosyl-transferase gene (*CsUFGT*) in tea positively regulates the accumulation of anthocyanin, resulting in the purple-leafed Jinmingzao tea (Chen et al., 2020). In strawberries, a gene mutation in the upstream regulatory region of *FnMYB10* results in the white fruit phenotype of *Fragaria nilgerrensis* (Zhang et al., 2020). In this study, a wild *D. cirrhosa* population with varying tuber colors (light red, red, dark red, and brownish-red) was obtained from a natural habitat in southern China in an effort to reveal the genetic mechanism of the pigment change in its tubers and build up our knowledge of this plant to better utilize this wild germplasm resource.

Flavonoids such as naringin, anthocyanin, and proanthocyanin (PA) share the phenylpropane and flavonoid pathways. The genes and enzymes involved in this pathway have been studied in depth (Zhao, Pang & Dixon, 2010; Marles, Ray & Gruber, 2003). PAs are oligomeric and polymeric end products of the flavonoid pathway that are derived from flava-3-ols and synthesized by the enzymes encoded by a series of structural genes. The upstream enzymes chalcone synthase (CHS), chalcone isomerase (CHI), and dihydroflavonol-4-reductase (DFR) work together to synthesize the downstream products leucoanthocyanidin and cyanidin (Aron & Kennedy, 2010; Tanner et al., 2003). Because of this, the further synthesis of PA polymers requires leucoanthocyanidin reductase (LAR), anthocyanidin synthase (ANS), and anthocyanidin reductase (ANR) to catalyze leucoanthocyanidin and cyanidin to catechin and epicatechin, respectively. Then, two or three polymer units linked together generate dimer and trimer PAs, while three or more polymer units linked together generate oligomeric PAs, which finally accumulate in the vacuole and polymerize (Gong & Pegg, 2017; Zhao, Pang & Dixon, 2010). The distribution and degree of the polymerization of PA vary with plant species and tissue parts. In pecans, the most abundant PAs are dimer and trimer polymers (Zhang et al., 2018). In *Arabidopsis thaliana* (Arabidopsis), catechins reach their maximum levels at the mid- to late torpedo stage of embryogenesis (Kleindt et al., 2010). PAs are abundantly available in the fruits, leaves, and seeds of plants (Dixon, Xie & Sharma, 2005; Wan et al., 2020), and insoluble brown or reddish-brown pigments form when the organs mature (Marles, Ray & Gruber, 2003). In addition, many studies have demonstrated the bioactivity and health-protecting functions of PAs (Santos-Buelga & Scalbert, 2000; Luximon-Ramma et al., 2002). Recent clinical experiments have revealed the curative effect of PAs in the treatment of neurological disorders and cardiac injury (Wang et al., 2012a; Wang et al., 2012b; Sadek et al., 2021). The beneficial functions of PAs in human health also include scavenging free radicals and preventing cardiovascular disease and cancer (Bagchi et al., 2000; Soobrattee, Neergheen & Luximon-Ramma, 2005). Thus far, no transcriptome data is available for *D. cirrhosa*, and the genes regulating flavonoid/PA biosynthesis are still unclear.

Transcription factors (TFs) have recently been recognized for their crucial roles in the regulation of flavonoid biosynthesis (Martin & Paz-Ares, 1997). Transcription factors of the MYB-bHLH-WD40 (MBW) complex play crucial roles in flavonoid accumulation (Xu, Dubos & Lepiniec, 2015; Feller et al., 2011). The overexpression of *MdMYB3* in red apples can increase pigmentation and activate the accumulation of high levels of anthocyanins and flavonols (Vimolmangkang et al., 2013). In Arabidopsis, the loss of *MYBL2* activity was found to promote the accumulation of anthocyanin and proanthocyanin (Dubos et al., 2010). *WRKY2/34-VQ20* complexes negatively regulate the expression of related MYB TFs during pollen development in *A. thaliana* (L.) (Lei, Ma & Yu, 2018). The deregulated expression of *SiMYB* 12 leads to a pink fruit color in tomatoes (Meng, Zhao & Ding, 2020). The transcriptional regulation of flavonoid/PA biosynthesis is complex, and numerous TFs participate in this biosynthetic process. Thus far, little is known about the regulators involved in the PA biosynthesis pathways of *D. cirrhosa*.

As a natural dye and medicinal plant, the pigment accumulation in *D. cirrhosa* tubers makes this species valuable (Yang, Guan & Tang, 2018). Although numerous studies have been conducted on flavonoids, and the PA metabolism pathway has been clearly elucidated in model plants (Tohge, Nishiyama & Hirai, 2005), the flavonoid biosynthesis and PA-related regulators in *D. cirrhosa* tubers remain unknown. Therefore, it is particularly crucial to reveal the mechanism of color formation in this species. In this study, metabolome and transcriptome analyses were integrated to profile the putative flavonoid pathway and reveal the functions of genes related to color formation in *D. cirrhosa*. The findings will provide a foundation for further study of the genetic engineering and breeding.

## Materials & Methods

### Plant material collection

**Figure 1:**
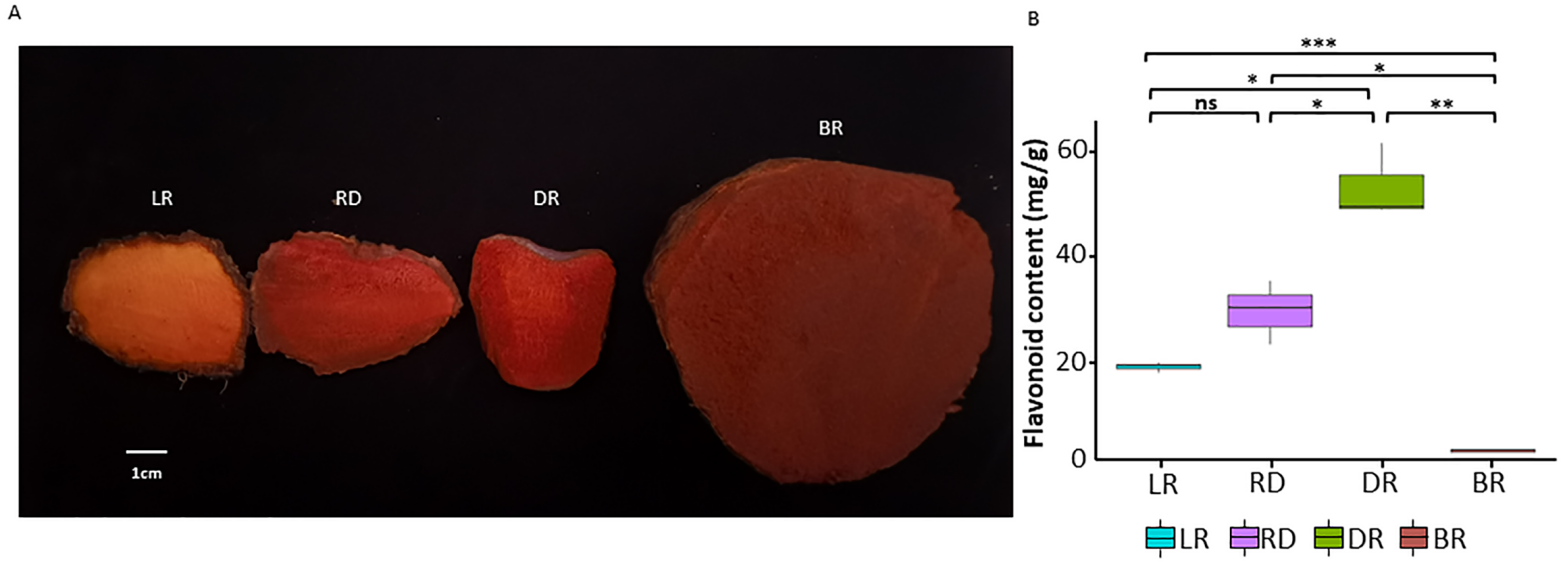
The tuber differences in four stages. (A) Four wild *Dioscorea cirrhosa* tubers with different colors. (B) Total flavonoid content in four groups. Three biological replicates are shown. Each color represents one tuber, an asterisk (*) indicates significance level and ns indicates non-significant.

A total of four tuber types with distinct colors were obtained from their natural habitat located in the hilly region of Shunde, Guangdong Province, China (112.65°E, 22.88°N). All samples were collected from the same location. The color gradient of the four tubers was easily visible (Fig. 1A). Therefore, four differently colored tubers with three independent biological replicates (12 samples in total) were used in this study. The tuber flesh was flash frozen in liquid nitrogen and kept at −80 °C for subsequent experiments.

### Determination of total flavonoid content

The detection of total flavonoid content was carried out using the colorimetric aluminum nitrate method (Moreno et al., 2000). The tubers were dried and powdered, then 1.0 g of each sample was accurately taken, mixed with 30 ml of 60% ethanol, extracted ultrasonically for 40 min, followed by suction filtration and centrifugation. A total of 0.1 ml of extract from each sample was pipetted into a burette, then 0.5 ml of 5% NaNO_2_ solution, 0.5 ml of 10% Al (NO_3_)_3_ solution, and 4 ml of 4% NaOH solution were then respectively added. The volume was adjusted with 60% ethanol to 10 ml, shaken, and then the absorbance value was measured at 510 nm. The total flavonoid content was calculated using rutin as the standard (Chen, Wang & Wan, 2010). A significance analysis of the total flavonoid content between groups was performed using the *t*. test (version 4.0.3) package in R.

### Metabolite identification and quantification

Widely-targeted metabolite profiling analyses were performed by Metware Biotechnology Co., Ltd. (Wuhan, China). The identification and quantification of the metabolites were performed as described previously, using the LC-ESI-MS/MS system (UPLC, Ultra Performance Liquid Chromatography; Shimpack UFLC SHIMADZU CBM30A; MS/MS, Applied Biosystems 4500QTRAP) (Dong et al., 2014). Using the MWDB database (Metware Database, http://www.metware.cn), the mass spectrometry data of all samples were obtained and processed using Analyst (software version 1.6.3).

### Metabolomic data analysis

A principal component analysis (PCA) was conducted to understand the overall differences between samples in each group as well as the variability between samples (Chen et al., 2009; Eriksson et al., 2006). The hierarchical cluster analysis (HCA) method was used to show the accumulation pattern of different metabolites in the four groups, and the R package heatmap was used to draw the cluster heatmap. The OPLS-DA method was used to maximize the metabolomic differences between the pairs of samples (Thévenot et al., 2015), and the variable importance in projection (VIP) value was used to calculate the relative importance of each metabolite. The PCA and OPLS-DA were both performed using R (software version 4.0.3; R Core Team, 2020). Metabolites with a fold change ≥ 2 and a fold change ≤ 0.5 were selected, and metabolites with a VIP > 1 were considered differential metabolites for group discrimination (Mizoi, Shinozaki & Yamaguchi-Shinozaki, 2012). The K-means analyses were performed in R.

### RNA-sequencing, data processing, and annotation

The total RNA isolation was conducted, as previously described, with three biological replicates prepared for each tuber type. The mRNA library construction and sequencing of each sample were performed in the Illumina Hiseq platform using the paired-end reads method. Then, clean reads were obtained by processing the raw reads and removing the low-quality bases, undetermined nucleotides, and adapter sequences. Trinity (software version 2.6.6) (Grabherr, Haas & Yassour, 2011) was used to assemble clean reads, and the longest clusters were obtained using Corset hierarchical clustering (version 1.07) (Davidson & Oshlack, 2014), with soft clusters regarded as unigenes. The gene saturation was calculated and the gene expression level was measured in each sample using reads per kilobase per million (RPKM) value (Wang et al., 2012a; Wang et al., 2012b; Pertea et al., 2015).

To further investigate the functions of unigenes, the assembled unigenes were annotated by the following databases: non-redundant NCBI protein database (NR) (ftp://ftp.ncbi.nih.gov/blast/db) (Deng et al., 2006), Clusters of Orthologous Groups of proteins (KOG) (http://www.ncbi.nlm.nih.gov/COG) (Tatusov et al., 2001), a manually annotated and reviewed protein sequence database (SwissProt) (http://www.gpmaw.com/html/swiss-prot.html) (Boeckmann et al., 2003), Gene Ontology (GO) (http://geneontology.org/) (Ashburner et al., 2000), and the Kyoto Encyclopedia of Genes and Genomes (KEGG) (https://www.kegg.jp) (Minoru et al., 2008). BLAST (software version 2.10) (Altschul et al., 1990; Camacho et al., 2009) was used to align the sequences to the database and extract the pathways.

### Differentially expressed genes (DEGs) analysis

The DEGs analysis was performed using the DEGseq (version 1.30.1) (Varet et al., 2016) package in R. The Benjamini–Hochberg method was used to perform a multiple hypothesis test correction on the hypothesis test probability *p*-value <0.05 to obtain the false discovery rate (FDR). Differential gene screening was based on the threshold of —log_2_Fold Change— ≥ 1 and FDR < 0.05 (Love, Huber & Anders, 2014). To compare the differences in DEGs among samples, we calculated the correlation of samples and drew the expression heatmap based on the Pearson’s correlation coefficient. A gene counting analysis was implemented by featureCounts (version 1.6) (Liao, Smyth & Shi, 2013). Furthermore, the identified DEGs were subjected to an enrichment analysis using GO and KEGG annotations and a pathway analysis using the clusterProfile (version 1.6) R package.

### Co-expression network construction

To investigate the gene modules associated with pigment accumulation and PA synthesis, a weighted gene co-expression network analysis (WGCNA) was performed using the WGCNA (version 1.31) (Langfelder & Horvath, 2008)) package in R. Network construction and module identification were conducted using the topological overlap measure (TOM). Gene modules were calculated based on the default settings with minModuleSize = 30, mergeCutHeight = 0.25, and power = 14. The eigengene value, total connectivity and intramodular connectivity, KME (eigengene-based connectivity), and *p*-values were calculated for the genes in each module, and the correlation coefficients among modules were used to construct the expression network.

## Results

### Comparison of tuber coloration

To investigate pigment accumulation in *D. cirrhosa*, we collected tubers at four color stages: LR (light red), RD (red), DR (dark red), and BR (brownish-red). Obvious color differences could be observed in these four tubers (Fig. 1A). Flavonoids are the key substances that affect the phenotypic color changes of plants (Wang et al., 2019a; Wang et al., 2019b). In this work, total flavonoid content was detected in the tubers on the basis of three biological replicates (Table S1). The results showed that the total flavonoid content reached the highest level in DR and then declined to the lowest level in BR. The *t*-test analysis revealed that 5 out of the 6 groups had significant differences in flavonoid content (Fig. 1B). The results showed that the content of flavonoids was significantly different in the pairwise comparison, indicating those samples could be used for further analysis.

### Identification of differentially accumulated metabolites (DAMs) in the tubers

**Figure 2:**
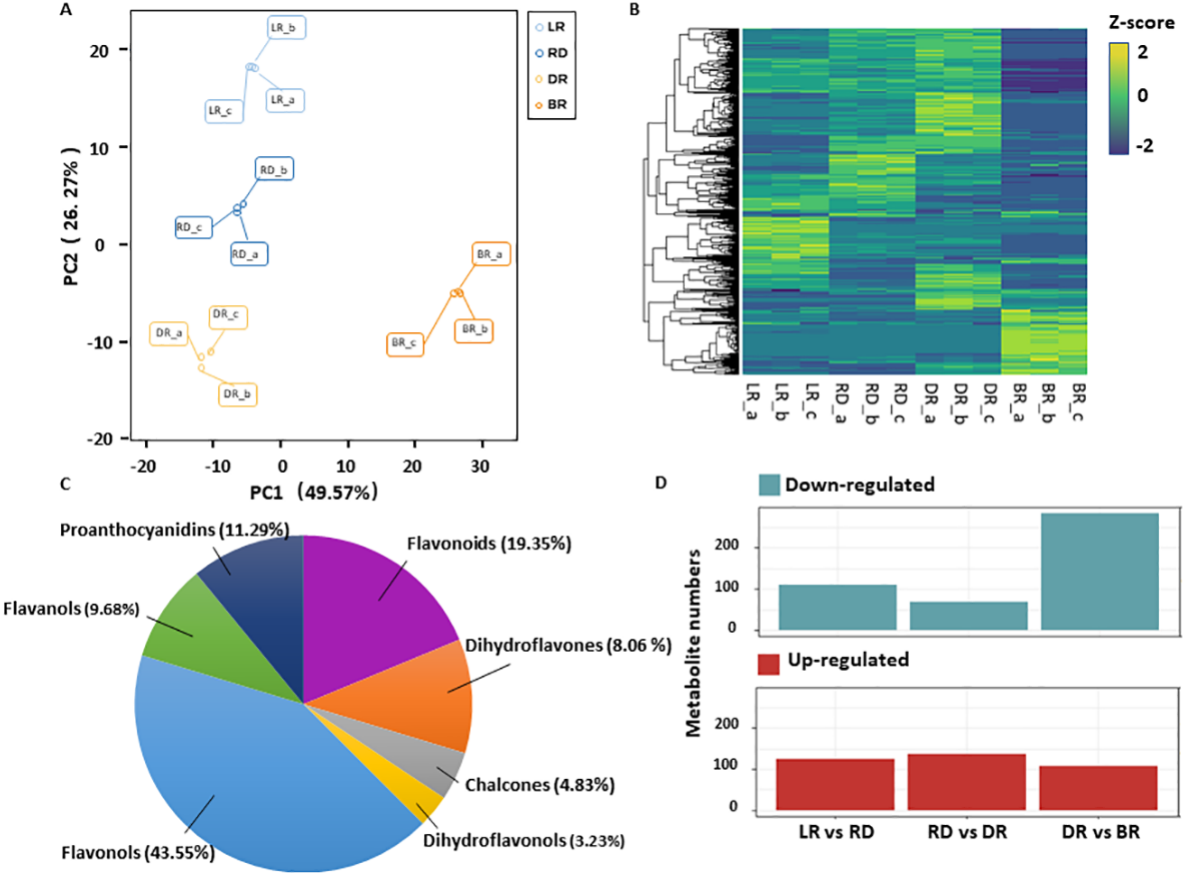
Differential metabolite analysis. (A) Principal component analysis (PCA) score plot of metabolites in different groups. The *x*-axis represents the first principal component and the *y*-axis represents the second principal component. (B) Hierarchical cluster analysis (HCA) plot. Each column represents a sample and each row represents a metabolite. The different colors in the clustering heat map represent the different contents of metabolites. (C & D) The quantitative distribution of DAMs and differentially up-regulated and down-regulated metabolites among the three groups (LD *vs* RD; RD *vs* DR; and DR *vs* BR), respectively.

A total of 531 metabolites were detected based on the UPLC MS/MS detection platform, primarily divided into 16 categories including: amino acids and their derivatives, flavonoids, alkaloids, phenolic acids, lipids, nucleotides and their derivatives, fatty acids, lignans, and steroids (Table S2). Among these, 62 flavonoid metabolites (FMs) were identified in this study: 12 flavonoids, 27 flavonols, five dihydroflavones, six flavanols, three chalcones, two dihydroflavonols, and seven proanthocyanidins (Fig. 2C, Table S3). To compare the overall metabolite differences among the groups and the variability between the intra-group samples, the metabolomic dataset obtained was subjected to a principal component analysis (PCA). The results showed that these groups were clearly separated in score, with 48.9% of the first principal component (PC1) and 25.25% of the second principal component (PC2) (Fig. 2A). The heatmap hierarchical clustering showed that the metabolite contents in LR, RD, DR and BR varied greatly (Fig. 2B). These results indicate that the metabolites of the four groups differ significantly and that the metabolomic data were highly reliable.

The orthogonal partial least squares-discriminant analysis (OPLS-DA) method was used to find different metabolites between groups by calculating the correlation between components and by removing irrelevant difference information (Boccard & Rutledge, 2013). A volcano map was used to show the OPLS-DA results (Fig. S1). Based on the threshold of VIP (variable importance) >1 and fold change ≥ 2 or ≤ 0.5, a total of 467 DAMs were identified in the comparison groups. Among these, 234 DAMs were detected between LR and RD (124 up- and 110 down-regulated), 205 DAMs were detected between RD and DR (136 up- and 69 down-regulated), and 390 DAMs were detected between DR and BR (87 up- and 303 down-regulated), respectively (Fig. 2D, Table S4). The results indicate that the DR-BR period may be a crucial turning point for metabolite down-regulation. Conversely, the RD-DR period may be a pivotal point for metabolite up-regulation. Additionally, a KEGG analysis was also performed to explore the functional classification of the DAMs obtained from each comparison group. Except for the RD *vs.* DR group, the other five groups were found to possess the “flavonoid biosynthesis” KEGG pathway. “Phenylpropane biosynthesis” was enriched in the LR *vs.* RD, DR *vs.* DR, LR *vs.* DR, and RD *vs.*. BR groups, and “flavone and flavonol biosynthesis” was enriched in DR *vs.* BR, LR *vs.* BR, and RD *vs.* BR (Table S5, Fig. S2). These results indicate that flavonoid biosynthesis may play an important role in the color formation of tubers.

### Dynamic trends and patterns of DAMs

**Figure 3:**
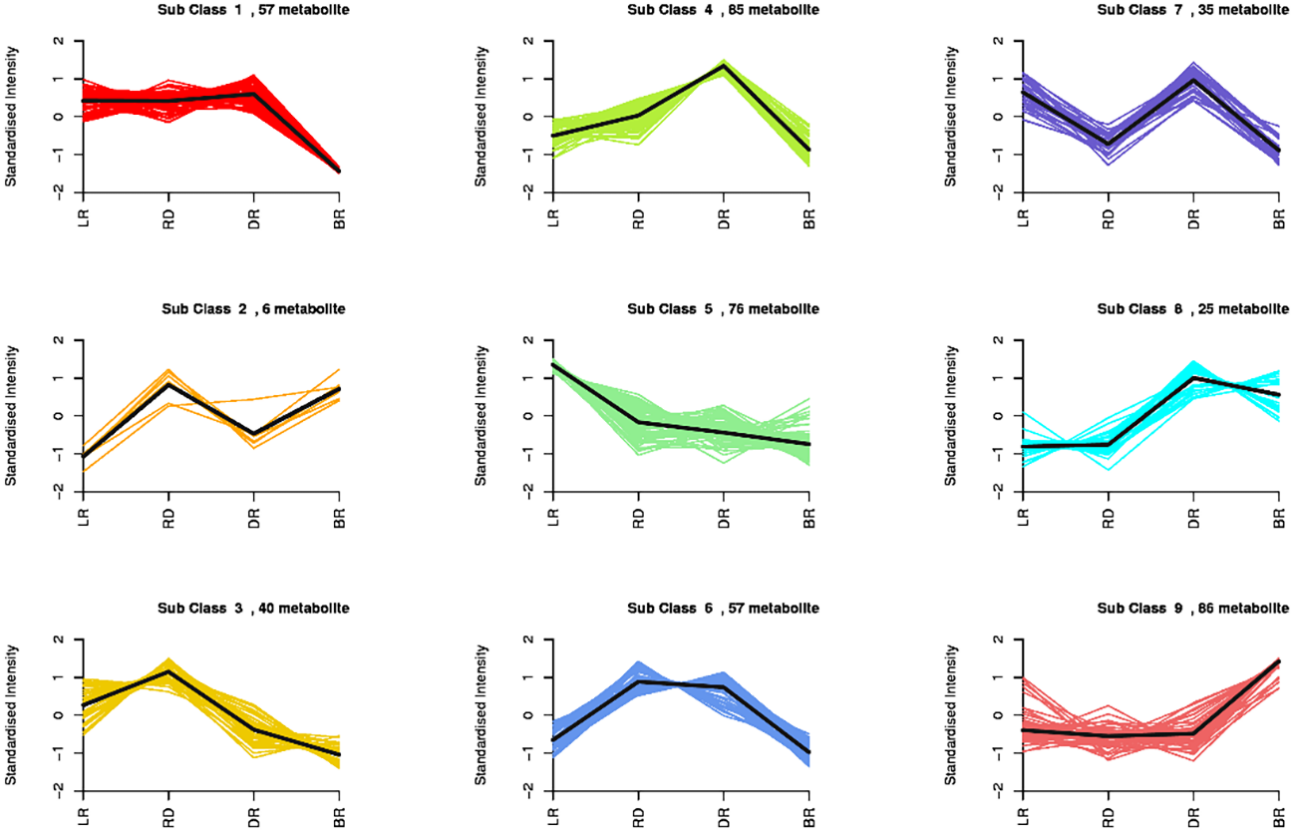
Nine metabolite clusters with distinct change patterns. The metabolites were scaled using *Z*-score of relative content (mean value of three replications).

By using a K-means analysis, 467 DAMs were divided into nine clusters with similar dynamic and change patterns (Fig. 3, Table S6). Among these, class 4 and class 9 harbored the greatest number of metabolites. The class 4 metabolites gradually increased from LR to DR, then decreased in BR, which was consistent with the change of total flavonoid content, primarily including phenolic acids, amino acids and derivatives, phenolamine, organic acids, saccharides, and flavonoids. Noticeably, only five flavonoids (pinocembroside, 3′-O-Methyl-(-)-epicatechin, gallocatechin, epicatechin-epiafzelechin, and persicoside) were assigned to class 4; these flavonoids may play an important role in tuber color formation. The metabolites in class 9 significantly accumulated in BR, including 12 flavonoids, 10 amino acids, and eight phenolic acids. These results showed that the flavonoids distributed in class 9 may be important metabolites that affect the color formation of BR tubers whereas metabolites in class 4 may affect the color formation of LR, RD and BR tubers.

### FM difference in the tubers during color formation

**Figure 4:**
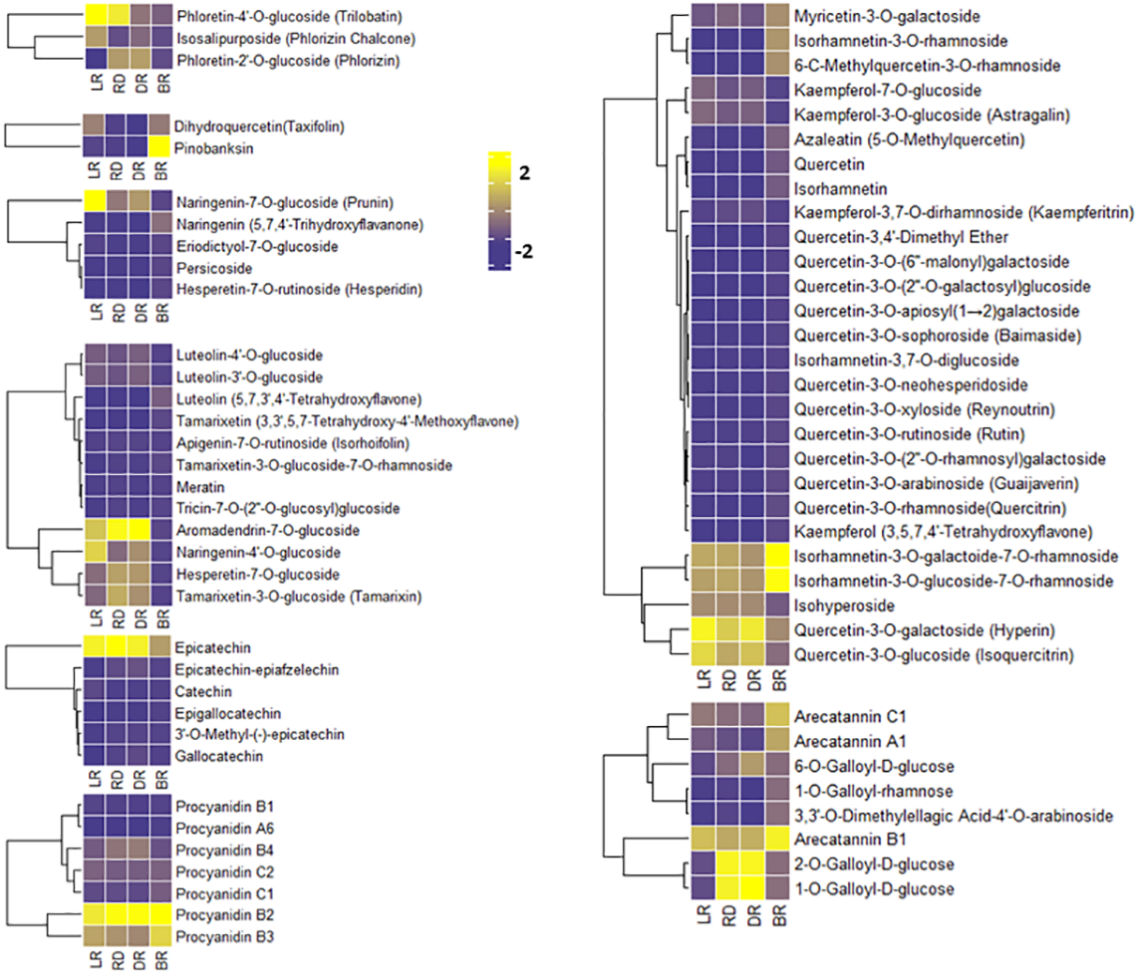
Varying levels of flavonoid metabolites in tubers. (A) Heatmap of chalcone content in LR, RD, DR, and BR. (B) Heatmap of dihydroflavonol content in LR, RD, DR, and BR. (C) Heatmap of flavonoid content in LR, RD, DR, and BR. (D) Heatmap of flavanol content in LR, RD, DR, and BR. (E) Heatmap of dihydroflavone content in LR, RD, DR, and BR. (F) Heatmap of PA content in LR, RD, DR, and BR. (G) Heatmap of flavonol content in LR, RD, DR, and BR. (H) Heatmap of tannin content in LR, RD, DR, and BR. The metabolites were scaled using *Z*-score of relative content (mean value of three replications) in the heatmap.

To compare the FM changes in tubers, a heatmap was used to display the varying levels of these compounds. A considerable portion of FMs increased from LR to DR and occurred at high levels in DR, whereas they declined to low levels in BR, including phloretin-4′-O-glucoside, aromadendrin-7-O-glucoside, naringenin-7-O-glucoside, and quercetin-3-O-galactoside (Fig. 4). Only seven FMs were found in BR tubers: naringenin, tamarixetin, luteolin, kaempferol, quercetin-3,4′-dimethyl ether, quercetin, and azaleatin (Fig. 4, Table S3). These upstream substances all exhibited the highest levels in BR compared to the other three groups. In contrast, the downstream metabolites, primarily luteolin-4′-O-glucoside, luteolin-3′-O-glucoside, naringenin-7-O-glucoside, kaempferol-3-O-glucoside, kaempferol-3,7-O-dirhamnoside, quercetin-3-O-glucoside, and quercetin 3-galactoside, exhibited the lowest levels in BR (Fig. 4). These results indicate that BR has more upstream substrates to produce downstream flavonoids, and that these FMs may be associated with the brownish color formation in BR. This may be because the synthesis pathway of downstream metabolites in BR was blocked, resulting in the accumulation of upstream metabolites.

PAs are downstream metabolites of the flavonoid biosynthesis pathway (Taruscio, Barney & Exon, 2004). The PAs detected in this study included procyanidin A6, procyanidin B1, procyanidin B2, procyanidin B3, procyanidin B4, procyanidin C1, and procyanidin C2, catechin, epicatechin, and their derivatives (Fig. 4). It is noteworthy that both epicatechin and procyanidin B2 showed high levels in all groups, while procyanidin B3 only exhibited high levels in BR (Fig. 4). In addition, eight tannins were detected in total. The results showed that 1-O-Galloyl-D-glucose and 2-O-Galloyl-D-glucose were expressed at high levels in RD and DR, while arecatannin B1 showed the highest levels in BR (Fig. 4, Table S3). These findings suggest that these PAs may the key metabolites leading to color formation in tubers, and the tannins primarily accumulated in the later stages of tuber color formation.

### Transcriptome analysis

A total of 12 cDNA libraries were generated, and 33.3 Gb of raw bases were produced. High-quality clean reads were generated by removing low-quality reads. The Q30 content of the reads was more than 90%, and the GC content was 45%. Further de novo assembly yielded a total of 131,334 unigenes for analysis, with a mean length of 1,172 bp and an N50 length of 1,863 bp (Table S7). The annotation results of seven public databases showed that 88,183 (67.14%) unigenes were annotated in NR, 70,409 (53.61%) were annotated in GO, 66,135 (50.36%) were annotated in KEGG, 55,987 (42.63%) were annotated in KOG, and 61,518 (46.84%) were annotated in SwissProt. These results indicate that the RNA-seq assembled in this study were reliable for further analysis. Prior to this study, little genomic information was available for *D. cirrhosa*. This work provides the genomic data for *D. cirrhosa*.

### Functional annotation and enrichment analysis of DEGs

**Figure 5:**
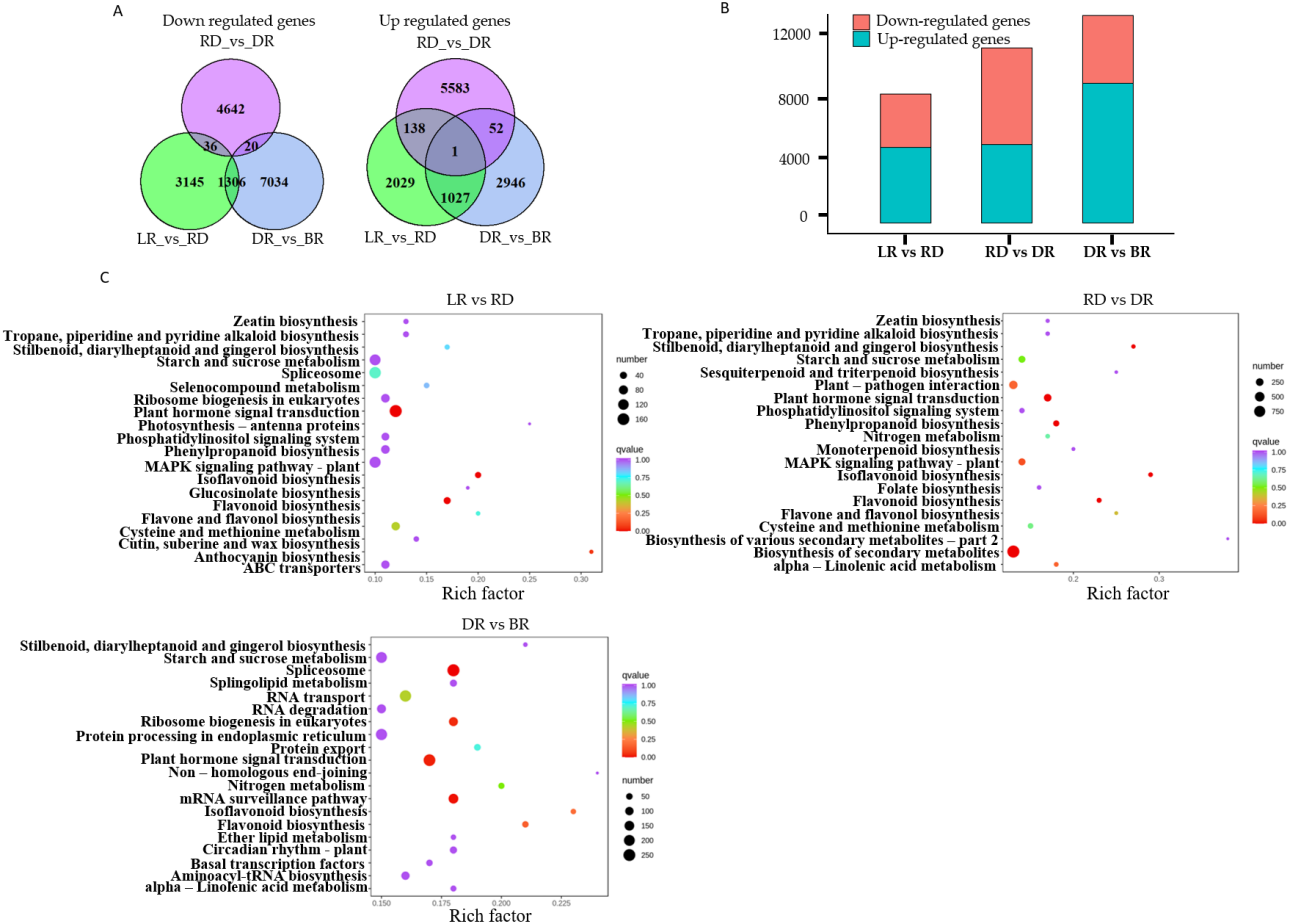
The differential gene analysis. (A) The Venn diagrams displaying the differentially expressed gene (DEG) overlap identified between groups. The numbers show up-regulated and down-regulated genes between groups. (B) The bar plot indicates up- and down-regulated DEGs identified between groups. (C) KEGG analysis in LR *vs* RD, RD *vs* DR, and DR *vs* BR; the color and size indicate the *p*-value and the number of DEGs, respectively.

A total of 22,865 DEGs were identified in this study. Among these, 3,195 down-regulated DEGs and 4,487 up-regulated DEGs were identified in LR *vs* RD, 5,574 down-regulated DEGs and 4,698 up-regulated DEGs were identified in RD *vs.* DR, and 4,026 down-regulated DEGs and 8,360 up-regulated DEGs were identified in DR *vs.* BR (Fig. 5A, Table S8). The number of up-regulated genes in DR *vs.* BR was significantly higher than down-regulated genes, whereas RD *vs* DR harbored more down-regulated genes than up-regulated genes (Fig. 5B). Thus, we concluded that the RD-DR and DR-BR periods were two important stages for pigment accumulation and color change.

Furthermore, a KEGG analysis was carried out to map the functions of the unigenes between groups. The results showed that plant hormone signal transduction, and flavonoid and isoflavonoid biosynthesis were the dominant biological process categories in the LR *vs.* RD period. Plant hormone signal transduction, isoflavonoid biosynthesis, and flavonoid biosynthesis were the top categories in RD *vs.* DR. In DR *vs.* BR, spliceosome, plant hormone signal transduction, mRNA surveillance pathway, ribosome biogenesis in eukaryotes, and flavonoid biosynthesis were the dominant categories (Fig. 5C). Studies have shown that hormone- and transcription-related genes both perform functions in flavonoid accumulation (Rai et al., 2016; Zheng et al., 2021a; Zheng et al., 2021b). In this work, we found that plant hormone signal transduction and flavonoid biosynthesis may play important roles in the color formation of *D. cirrhosa* tubers, and there may be a close relationship between these two biological processes in regulating PA synthesis in *D. cirrhosa*.

### Flavonoid biosynthesis pathways and related DEGs between the four groups

A total of 67 key candidate unigenes were found to be involved in PA biosynthesis by searching the KEGG database, including 13 enzymes that directly influence the flavonoid biosynthesis pathways (Table 1, Table S9). These regulated genes might be crucial for the synthesis of flavonoids in *D. cirrhosa*.

**Table 1:** Flavonoid pathway genes related to tuber color formation in *Dioscorea cirrhosa*.

Gene	Enzyme	KO id	No. All	DEGs in LR	DEGs in RD	DEGs in DR	DEGs in BR
PAL	phenylalanine ammonia-lyase	K10775	8	7	8	5	7
C4H	cinnamate 4-hydroxylase	K00487	2	2	2	2	2
4CL	4-coumarate-CoAligase	K01904	10	10	10	6	10
CHS	chalcone synthase	K00660	5	4	5	3	5
CHI	chalcone isomerase	K01859	8	7	8	6	8
F3H	flavonoid 3-hydroxylase	K00475	2	1	1	2	2
F3′H	flavonoid 3′-monooxygenase	K05280	5	5	5	4	5
F3′5′H	flavonoid 3 ′5′-hydroxylase	K13083	8	7	8	7	8
FLS	flavonol synthase	K05278	11	9	11	10	10
DFR	dihydroflavonol 4-reductase	K13082	2	1	1	1	2
ANS	anthocyanidin reductase	K05277	4	4	4	2	3
ANR	anthocyanidin synthase	K08695	3	3	3	3	3
LAR	leucoanthocyanidin reductase	K13081	1	1	1	1	1

**Figure 6:**
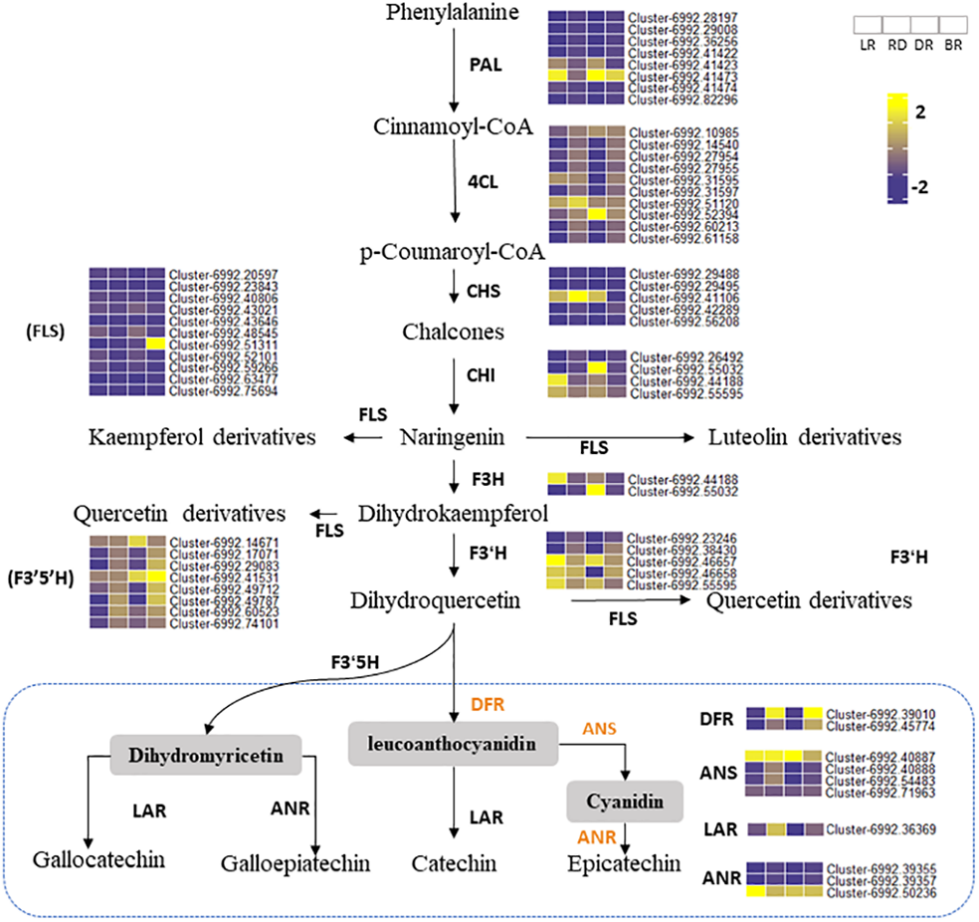
Putative flavonoid/PA pathways and DEGs in *Dioscorea cirrhosa*. PAL, phenylalanine ammonia-lyase; C4H, cinnamate 4-hydroxylase; 4CL, 4-coumarate-CoAligase; CHS, chalcone synthase; CHI, chalcone isomerase; F3H, flavonoid 3-hydroxylase; F3′H, flavonoid 3′-monooxygenase; FLS, flavonol synthase; F3′5′H, flavonoid 3′5′-hydroxylase; DFR, dihydroflavonol 4-reductase; ANS, anthocyanidin reductase; LAR, leucoanthocyanidin reductase; ANR, anthocyanidin synthase. The color scale represents the average of FPKM value (scaled using *Z*-score), yellow color indicate high expression and blue color indicate low expression.

Phenylalanine ammonia-lyase gene (PAL) is the first enzyme that catalyzes the conversion of phenylalanine to cinnamic acid (Ferrer et al., 2008). CHS is effective in flower color regulation (Chen et al., 2012). Flavonol synthase (FLS) genes control flavonol synthesis (Holton, Brugliera & Tanaka, 2010). CHI and FLS can catalyze dihydroflavonol to produce kaempferide, luteolin, and quercetin (Winkel-Shirley, 2001). In this study, the heatmap was conducted to show the gene expression levels among tubers (Fig. 6). Results showed that some PA-related genes only exhibited significantly higher expression in BR, including Cluster 6992.42289 (CHS), Cluster 6992.51311 (FLS), and Cluster 6992.42986 (CHI). This may explain why some metabolites (e.g., luteolin, naringenin, and quercetin) were only found in BR and were not detected in other tubers (Fig. 4). Notably, two PAL genes (Cluster 6992.41423 and Cluster 6992.47474) and one CHS gene (Cluster 6992.41106) showed high expression levels in LR, RD, and DR, but low expression levels in BR, which was consistent with the changes in flavonoid metabolites (luteolin-3-O-glucoside, luteolin-4-O-glucoside, aromadendrin-7-O-rutinoside, hesperetin-7-O-glucoside, and tamarixetin) (Fig. 4). ANS and ANR genes are the key regulators that catalyze cyanidin and dihydromyricetin to PAs, respectively, and DFR is the key enzyme for the catalysis of flavanol to leucoanthocyanidin (Pang et al., 2007; Xie, Sharma & Paiva, 2003). In this work, we found that three PA genes, including the DFR gene (Cluster 6992.39010), ANS gene (Cluster 6992.40887), and ANR gene (Cluster 6992.50236), showed consistently high expression patterns. This is consistent with the high level of PA metabolites found in tubers (epicatechin and procyanidin B2), indicating that these are the key genes for PA synthesis. In contrast, the expression levels of other PA genes showed no significance between groups. These results indicate that these genes are important in the synthesis of flavonoids and PA metabolites and that these may function together to regulate metabolite biosynthesis in *D. cirrhosa*.

### Co-expression network identified PA-related DEGs

**Figure 7:**
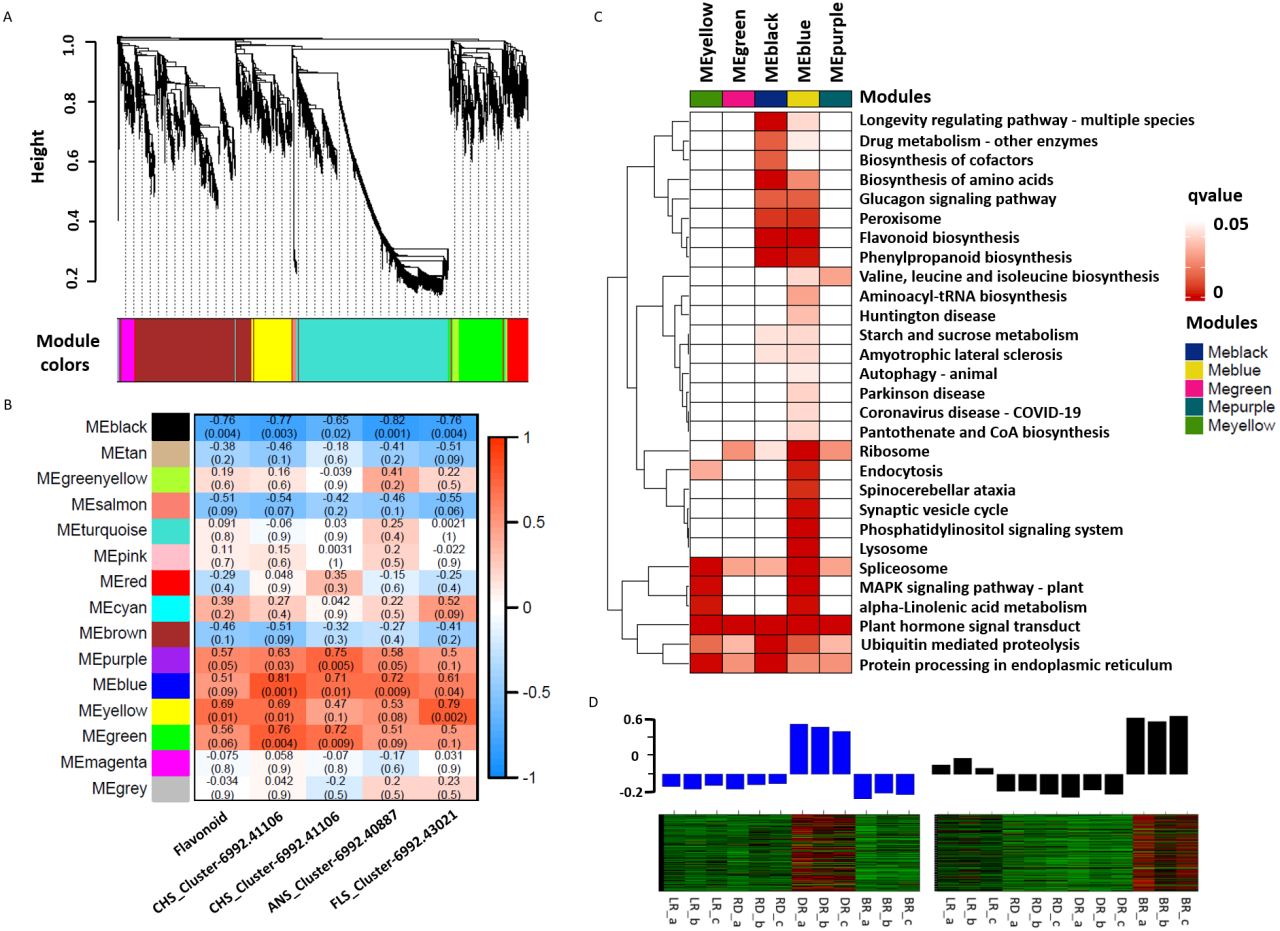
Weighted gene co-expression network analysis (WGCNA) of differentially expressed genes (DEGs) identified in tubers. (A) Hierarchical clustering tree of 7963 unigenes and 15 co-expression gene modules, with each module represented by the main tree branch. (B) Module-trait correlations and the corresponding *p*-values are in parentheses, each color represents one module. The color scale represents the module characteristics from −1 to 1. (C) KEGG enrichment analysis of the WGCNA modules; the KEGG terms with *p*-values < 0.05 are displayed. (D) Eigengene expression profile for blue and black module genes in samples. The bar graphs show the corresponding eigengene expression level.

WGCNA was used to reveal the correlation patterns of genes associated with PA biosynthesis. A co-expression network analysis was conducted based on the reads per kilobase per million (RPKM) value of 7,963 DEGs, resulting in 15 distinct modules, labeled with different colors, in which genes in the same modules had high correlation coefficients (Fig. 7A, Table S10). Furthermore, the total flavonoid content and the key genes related to PA biosynthesis were both used as trait data for the module-trait relationship analysis (Fig. 7B). Among the modules, MEblue had the highest correlation with PA biosynthesis and was related to ANS_Cluster-6992.41106 by the highest level of *r* = 0.81, *p* = 0.001. In contrast, MEblack had the lowest correlation with ANS_Cluster-6992.40887 by the lowest level of r = −0.81, *p* = 0.001. This indicated that a strong positive regulation and a negative regulation of PA synthesis genes were distributed in the MEblue and MEblack modules, respectively. Through calculating the eigengenes in these two modules, significantly high expression levels were exhibited in DR and BR (Fig. 7D). Further KEGG mapping of the modules with high correlation coefficients showed that MEblue harbored the most KEGG terms (Fig. 7C). It is worth noting that the MEblue and MEblack modules were both highly related to the biosynthesis of phenylpropanoids, flavonoid, amino acids, peroxisome, and the glucagon signaling pathway, while plant hormone signal transduction exhibited particularly high enrichment levels in each module, suggesting potential functions in the color formation process of *D. cirrhosa* tubers.

### Identification of hub TFs involved in pigment accumulation

**Figure 8:**
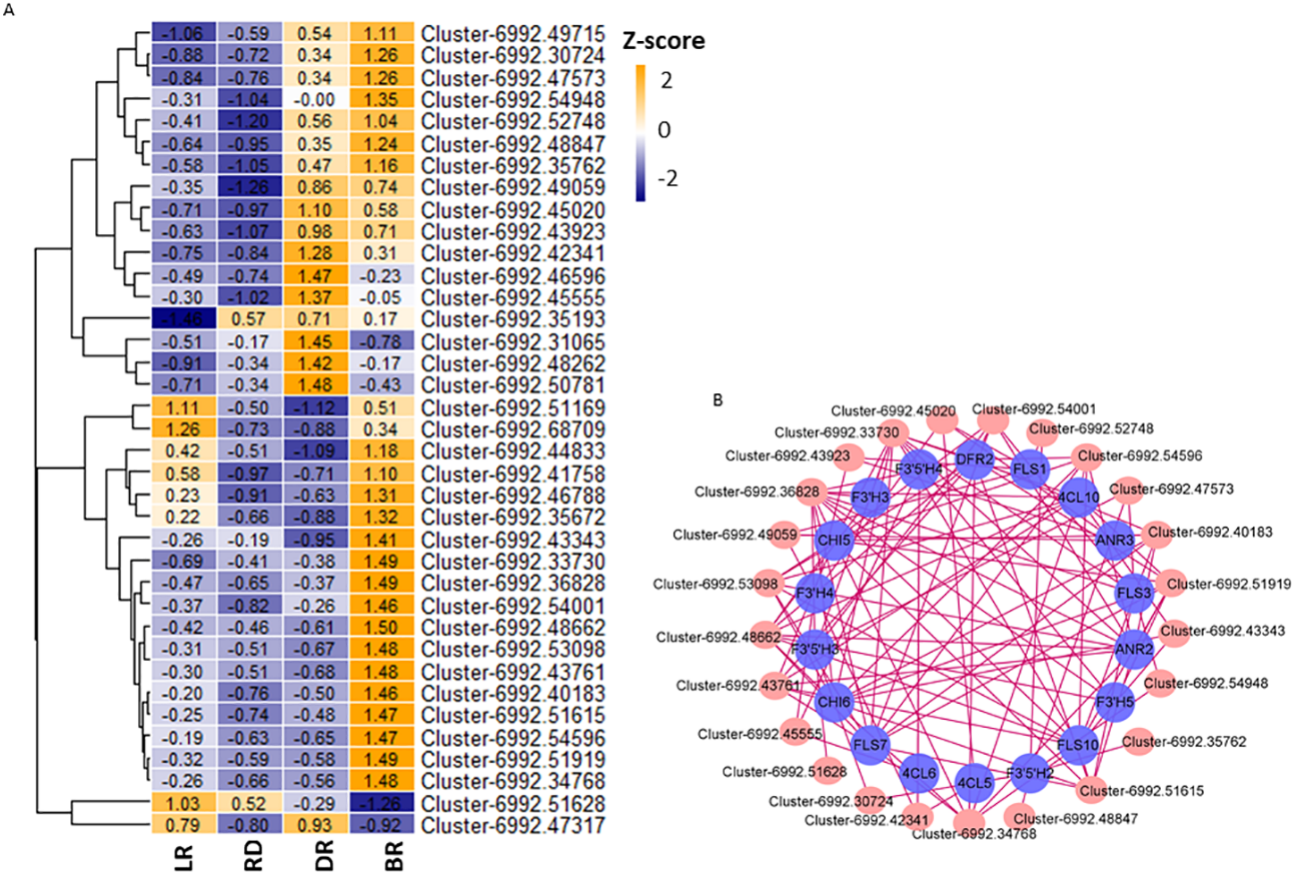
TFs related to flavonoid/homone biosynthesis. (A) Flavonoid/homone-related TFs expression levels. The color scale represents the average FPKM value (scaled using *Z*-score). The numbers in the heatmap indicate the *Z*-score value of each gene in the samples. (B) Regulatory networks of TFs and PAs genes, each pink circle represents hub TFs, and each blue circle represents flavonoid pathway genes.

Aux/IAA transcription factors and AP2/ERF-ERF transcription factors belong to plant hormone gene families, which regulate auxin and ethylene biosynthesis, respectively. They are involved in various biological functions, such as plant development, fruit and seed maturation, and disease resistance (Carole et al., 2013; Ying et al., 2010; Mizoi, Shinozaki & Yamaguchi-Shinozaki, 2012). To further study the correlation between TFs and flavonoid pathway genes in *D. cirrhosa*, we examined the expression patterns of the 37 candidate TFs with high expression levels in the samples. Among these, 11 were plant hormone pathway TFs (Fig. 8A, Table S11). Results showed that almost all TFs were significantly expressed in DR and BR. In comparation, gene expression levels in LR and RD were low, indicating that flavonoid and hormone biosynthesis were greatly enhanced in the late stages of tuber color formation (DR and BR). Pearson correlations were calculated between TFs and flavonoid genes. We used Cytoscape (version 3.8.2) (Shannon et al., 2003) to draw the gene connection network between 24 transcription factors and 18 flavonoid genes with high correlation (Fig. 8B). Among the TFs, Cluster-6992.36828 (AP2 TF) harbored the most edges related to the flavonoid pathway and was highly expressed in BR. Cluster-6992.45020 (AP2 TF) and Cluster-6992.48662 (MYB TF) harbored the second-most edges and showed high expression levels in the LR and BR tubers, respectively. The Ethylene RESPONSE FACTOR (ERF) and Aux/IAA transcription factor family genes harbored a number of edges with flavonoid genes, including Cluster-6992.45555 (AUX), Cluster-6992.53098 (bZIP), Cluster-6992.43923 (AP2), Cluster-6992.51615 (bZIP), and Cluster-6992.43923 (AP2). Notably, the DFR and ANR genes were also found to be highly associated with TFs, suggesting they may be key regulators in PA biosynthesis (Fig. 8B). These results indicate that the flavonoid TFs and hormone TFs may contribute to flavonoid/PA biosynthesis in *D. cirrhosa*.

## Discussion

As a relatively new field in the post-genomic era, plant metabolomics has been effectively applied to assess the changes of metabolites in different tissues, species or developmental stages (Kaiser, 2012; Li et al., 2018). In this study, a total of 531 metabolites, 62 of which were flavonoids, were identified using a widely targeted UPLC-MS/MS metabolomics approach. The pairwise comparison of differential metabolites showed that metabolites were primarily up-regulated in the early stages of color formation (LR, RD, and DR), and a considerable number of metabolites were down-regulated in the late stage of color formation (BR). Further, K-means clustering showed that the metabolites that increased from LR to DR and decreased in BR were mainly phenolic acids, amino acids, phenolamine, organic acids, saccharides, and flavonoids. A KEGG analysis of metabolites showed that these groups were significantly enriched in the pathways of flavonoid biosynthesis, phenylpropane biosynthesis, and flavone and flavonol biosynthesis. These results suggest that *D. cirrhosa* exhibits flavonoid metabolic activity during color formation, which is consistent with the results of the transcriptome analysis. Anthocyanins and proanthocyanins are the key flavonoids found in fruits, flowers, leaves, tubers, and other organs that affect the nutrition composition and color phenotype of plants (Chen et al., 2021; Zheng et al., 2021a; Zheng et al., 2021b; Wang, Chen & Wang, 2009). There were 13 total proanthocyanins identified in this work, and no anthocyanins were detected in *D. cirrhosa* tubers. Previous research has revealed that the anthocyanin biosynthesis pathways can be blocked by LAR and ANR enzymes and diverted into producing PAs instead of anthocyanins (Deluc et al., 2008), which may explain the absence of anthocyanins and the high content of PAs investigated in this work. Further research is needed to profile this result.

The key genes that regulate flavonoid synthesis in plants have been well studied in previous research (Durbin, Mccaig & Clegg, 2000). For example, the PAL gene is responsible for catechin production in tea (Wang et al., 2017). In purple sweet potatoes, the *IbANS* gene was confirmed to be associated with the anthocyanin biosynthesis activation (Zhou, Huang & Gong, 2010). The introduction of the apple *MdANR* gene into tobacco could inhibit the expression of the CHI and DFR genes, resulting in the decrease of PAs (Han et al., 2012). Through a transcriptome analysis, a total of 22,865 DEGs were identified as differentially expressed genes. Among these, 67 genes were identified as flavonoid-related genes. We found three genes that may be highly associated with PA biosynthesis: Cluster 6992.39010, Cluster 6992.40887, and Cluster 6992.50236, namely *dcDFR*, *dcANS*, and *dcANR*, respectively, which exhibit high expression levels during tuber pigment accumulation. Thus, these three genes may be the key genes regulating PA biosynthesis in *D. cirrhosa*. However, the effect of these genes on PA biosynthesis needs further verification.

In recent years, co-expression network analyses have become an effective way to reveal the internal relationship between genes and traits. Zheng et al. (2021a) and Zheng et al. (2021b) identified two modules, MEblue and MEbrown, which were highly correlated with PA biosynthesis. Previous research revealed that the grey60 module is associated with fruit ripening in strawberry plants (Li et al., 2021). In this study, we used WCGNA to characterize the genes obtained in *D. cirrhosa*, and found that the MEblue and MEblack modules were highly correlated with flavonoid biosynthesis. It was interesting that the genes in these two modules showed contrasting expression patterns in DR and BR tubers. These results suggest that the color changes of DR and BR were primarily regulated by the positive and negative genes in these two modules. Further, KEGG mapping revealed that the biosynthesis of secondary metabolites, plant hormone signal transduction, and flavonoid biosynthesis were the predominant biological processes in tubers, suggesting the potential role of these two pathways in the process of tuber color formation.

MYB, bHLH, and WRKY are key regulators that have been revealed to be strongly connected with PA accumulation in numerous plants (Wang et al., 2019a; Wang et al., 2019b; Li et al., 2019). In poplars, the overexpression of *MYB182* in hairy root culture and whole plants led to the decrease of PAs and anthocyanins as well as a reduction in the expression of flavonoid-related genes (Yoshida, Ma & Constabel, 2015). *VvMybPA2* genes ectopically expressed in grapevine hairy roots induced qualitative and quantitative changes in the PAs (Terrier, Torregrosa & Ageorges, 2009). In peaches, the *PpMYB7* gene is able to activate the PA-specific pathway gene *PpLAR1* (Hui et al., 2015). The over-expression of *MYBC1* and *WRKY44* in kiwifruit calli activated the expression of *F3*′5′*H* and PA-related biosynthetic genes, and increased the levels of PAs (Peng et al., 2020). Recent studies have demonstrated the important roles of phytohormones and hormone-related genes in the biosynthesis of the flavonoid pathway. For example, the AUX signal pathway may function along with the brassinosteroid and jasmonic acid (JA) synthesis signaling pathways to influence the accumulation of flavonoids in peanuts (Wan et al., 2020). Brassinosteroids have been shown to affect cytokinin (CK)-induced anthocyanin biosynthesis in Arabidopsis seedlings (Yuan, Peng & Zhi, 2015). Further research has shown that the F-box protein COI1 modulates JA-induced anthocyanin biosynthesis by mediating the ‘late’ anthocyanin biosynthetic genes DFR, LDOX, and UF3GT in Arabidopsis (Shan et al., 2009). In our study, 37 TFs that exhibited high expression levels and may participate in pigment regulation were investigated as potential regulators. Among these, 24 hub TFs exhibited high correlations with flavonoid-related genes. In addition to the MBW family gene, the plant hormone signal transduction-related TFs, including AP2, AUX, and bZIP TFs, also showed strong connections with flavonoid pathway genes, suggesting the flavonoid TFs and hormone TFs both play pivotal roles in flavonoid/PA biosynthesis in *D. cirrhosa*. Variations in the synthesis of genes and TFs will affect the pigmentation of fruits (Paauw, Koes & Quattrocchio, 2019). In our study, both the PA-related genes and TFs may affect the color formation of tubers. The regulation of flavonoid/proanthocyanin biosynthesis in *D. cirrhosa* tubers is worth further investigation.

## Conclusions

In this study, the various metabolites and flavonoid biosynthesis pathways in *D. cirrhosa* were revealed using widely targeted metabolome and transcriptome approaches. The 62 flavonoids in the four tubers were identified, and the levels of many PAs were found to be high. Three PA related genes with high expression patterns in the tubers were considered to be the key regulators of PA biosynthesis. A detailed analysis of DEGs found that the genes showed a higher expression pattern in the later tubers (DR/BR), and the two gene sets, MEblue and MEblack, exhibited significant positive and negative correlations with flavonoid biosynthesis, respectively. One new finding we present is that in addition to MYB, bHLH, and WRKY TFs, plant hormones and signal transduction related TFs were also highly associated with flavonoid genes, indicating their potential functions in tuber maturation and pigment accumulation. This is the first study utilizing metabolome and transcriptome analyses to investigate molecular mechanisms in *D. cirrhosa*. These data are a valuable resource for future research on gene functions that play a role in plant flavonoid biosynthesis and pigment changes in *D. cirrhosa*.

## Supplemental Information

10.7717/peerj.13659/supp-1 Supplemental Information 1 Total flavonoid content in tubers of *D. cirrhosa* (mg/g) Three biological replicates are shown.

10.7717/peerj.13659/supp-2 Supplemental Information 2 The metabolites detected in *D. cirrhosa* Relative content and information of 531 metabolites are shown.

10.7717/peerj.13659/supp-3 Supplemental Information 3 Flavonoids and tannins detected in samples Relative content and information of 62 flavonoids and 8 tannins.

10.7717/peerj.13659/supp-4 Supplemental Information 4 Differential metabolites between groups. Up/down regulated metabolites between groups of LR vs RD Up/down regulated metabolites between groups of LR vs RD, RD vs DR, and DR vs BR.

10.7717/peerj.13659/supp-5 Supplemental Information 5 KEGG analysis of metabolites between groups KEGG enrichment of 6 groups are shown, each table represents one group.

10.7717/peerj.13659/supp-6 Supplemental Information 6 K-means analysis results The 467 differential metabolites were divided into 9 sub classes.

10.7717/peerj.13659/supp-7 Supplemental Information 7 Summary of the RNA sequencing data

10.7717/peerj.13659/supp-8 Supplemental Information 8 DEGs identified between groups Differential gene analysis results of LR vs RD, RD vs DR, and DR vs BR.

10.7717/peerj.13659/supp-9 Supplemental Information 9 Genes related to flavonoid pathways Sixty-seven flavonoid-related genes and the RPKM value are shown.

10.7717/peerj.13659/supp-10 Supplemental Information 10 Module genes in co-expression network analysis Fifteen distinct modules labeled with different colors are shown, each table represents one module.

10.7717/peerj.13659/supp-11 Supplemental Information 11 Transcription factors investigated in this work The expression levels of 37 TFs identified in D. cirrhosa.

10.7717/peerj.13659/supp-12 Supplemental Information 12 Volcano map of differential metabolites between groups Red dots indicate up-regulated metabolites, and green dots indicate down-regulated metabolites.

10.7717/peerj.13659/supp-13 Supplemental Information 13 KEGG enrichment of differential metabolites between groups The color and size indicate the *p*-value and the number of DAMs, respectively.

## Additional Information and Declarations

### Data Availability

The following information was supplied regarding data availability: All raw data are available at NCBI SRA: PRJNA741609 and in the Supplementary Files.
