# Supplementary material for: Metabolome and transcriptome profiling reveal regulatory network and mechanism of flavonoid biosynthesis during color formation of Dioscorea cirrhosa L
Source: PeerJ. 2022 Jul 4;10:e13659. doi: 10.7717/peerj.13659 (PMC9261937; doi:10.7717/peerj.13659)
Supplement: Supplemental Information 12 — Red dots indicate up-regulated metabolites, and green dots indicate down-regulated metabolites. [file peerj-10-13659-s012.docx]

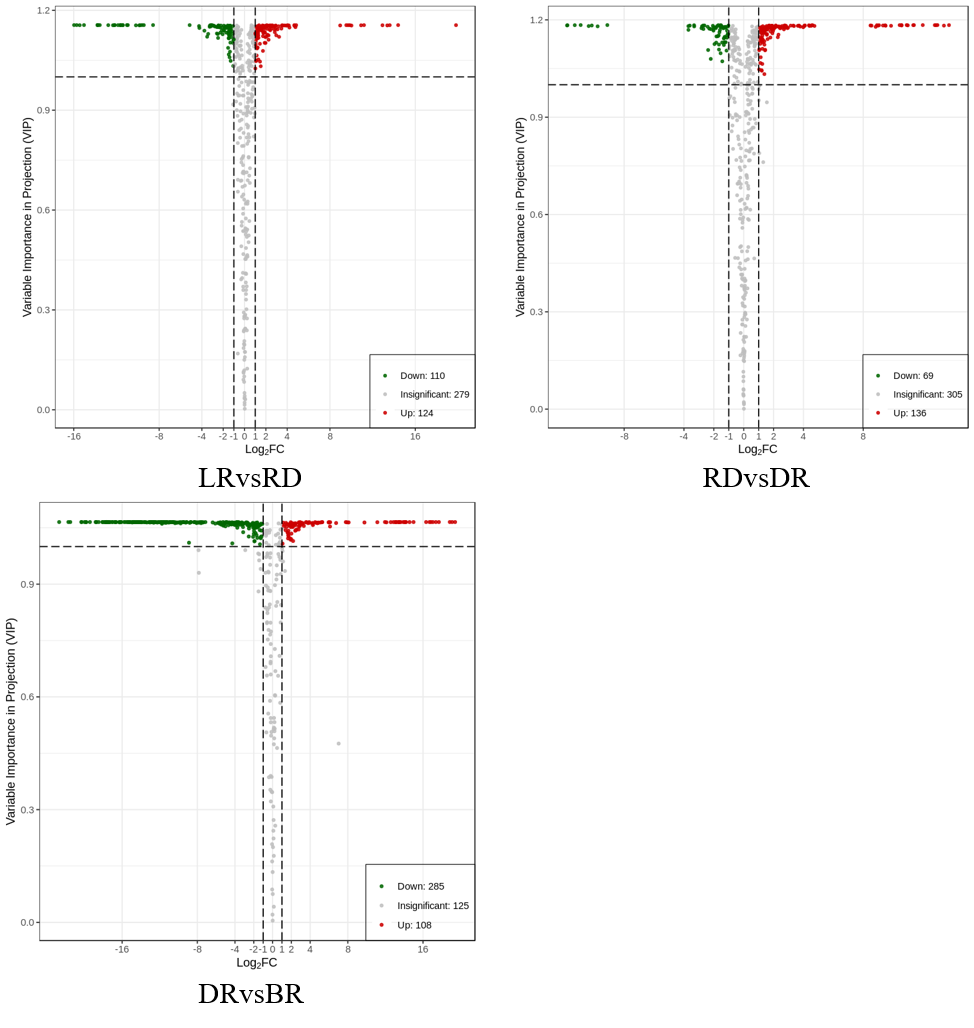


**Figure S1** **Volcano map of differential metabolites between groups.** Red dots indicate up-regulated metabolites, and green dots indicate down-regulated metabolites.
