# Supplementary material for: Metabolome and transcriptome profiling reveal regulatory network and mechanism of flavonoid biosynthesis during color formation of Dioscorea cirrhosa L
Source: PeerJ. 2022 Jul 4;10:e13659. doi: 10.7717/peerj.13659 (PMC9261937; doi:10.7717/peerj.13659)
Supplement: Supplemental Information 13 — The color and size indicate the p-value and the number of DAMs, respectively. [file peerj-10-13659-s013.docx]

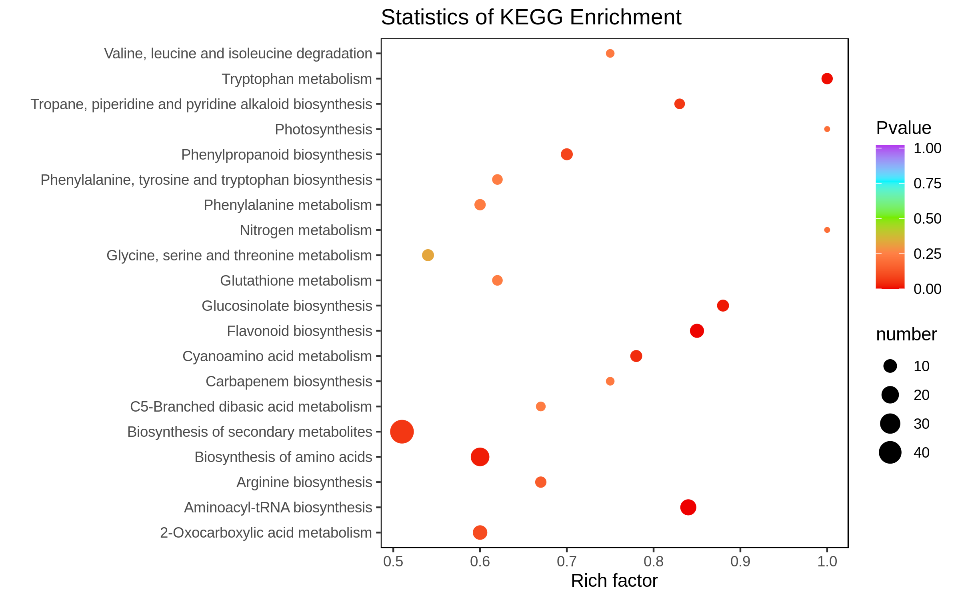


LR vs RD


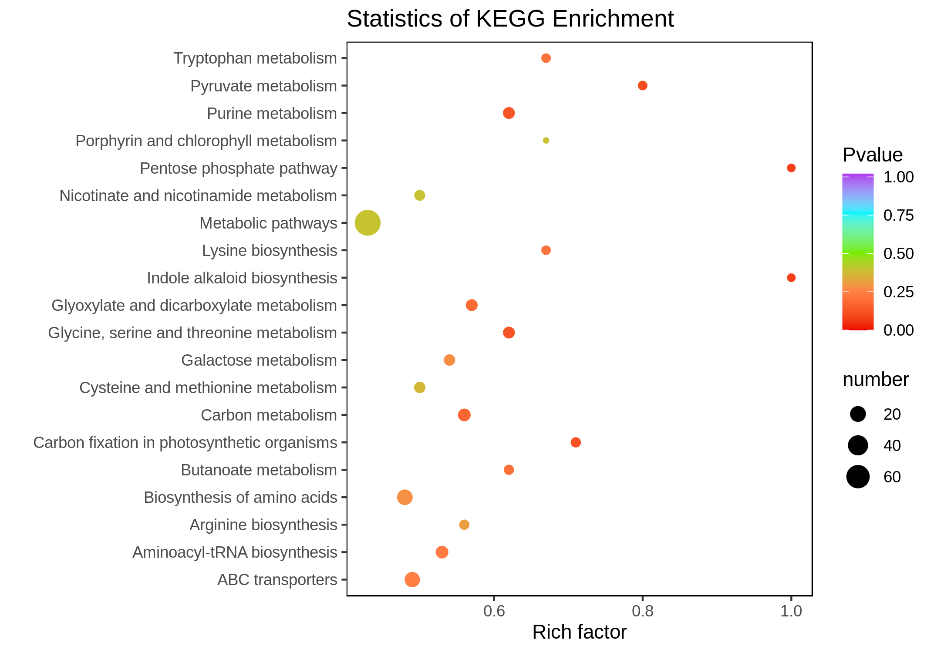


RD vs DR


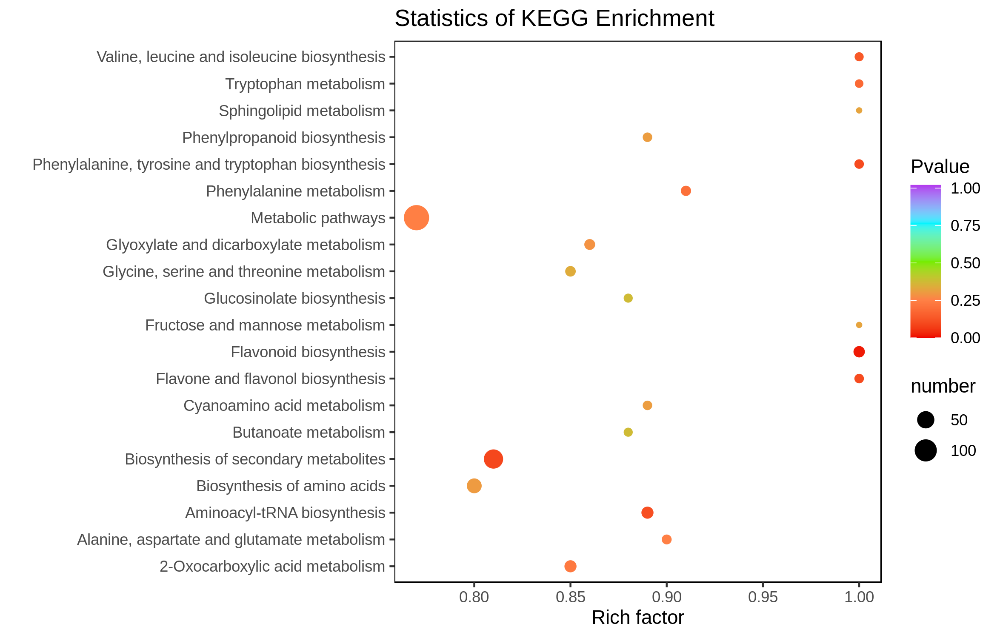


DR vs BR


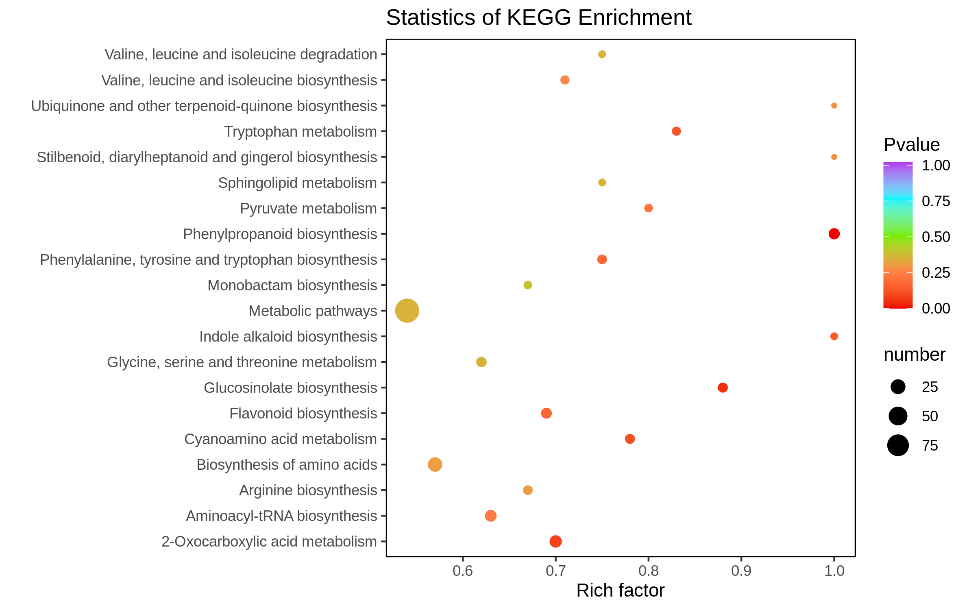


LR vs DR


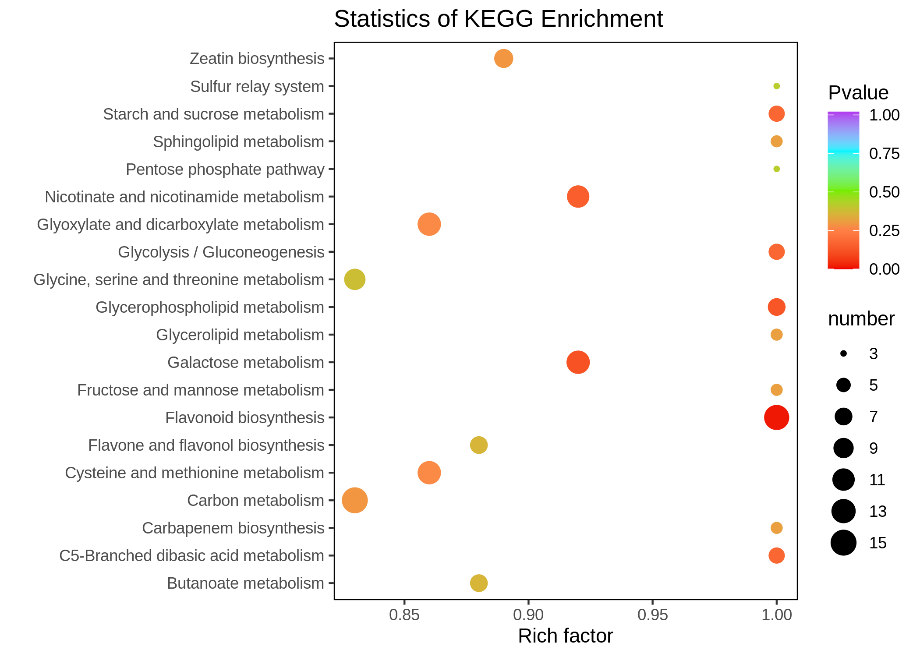


LR vs BR


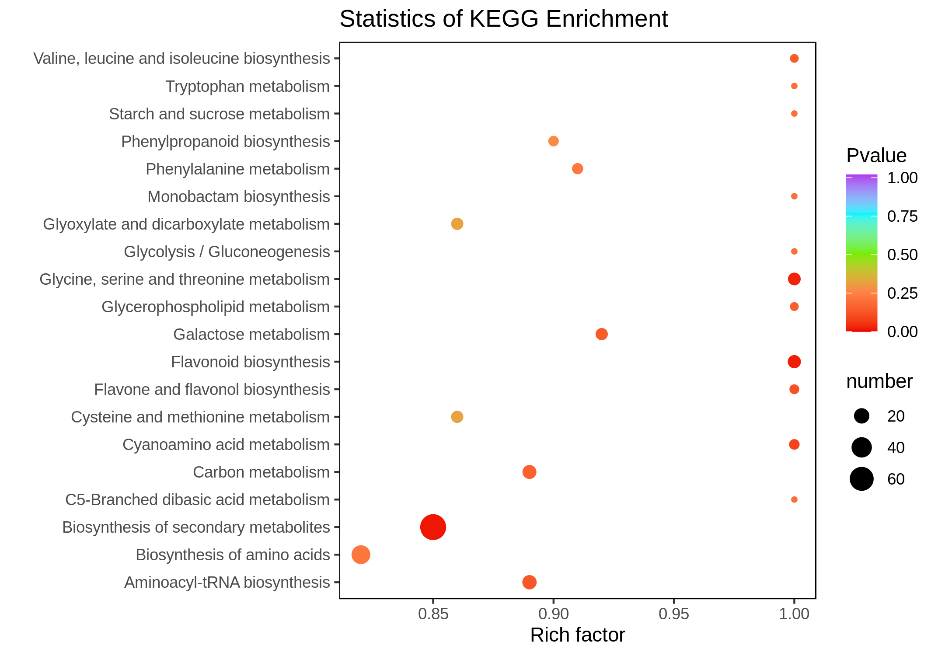


RD vs BR

**Figure S2** **KEGG enrichment of differential metabolites between groups.** The color and size indicate the p-value and the number of DAMs, respectively.
